# Recent Advances in Portable Dry Electrode EEG: Architecture and Applications in Brain-Computer Interfaces

**DOI:** 10.3390/s25165215

**Published:** 2025-08-21

**Authors:** Meihong Zhang, Bocheng Qian, Jianming Gao, Shaokai Zhao, Yibo Cui, Zhiguo Luo, Kecheng Shi, Erwei Yin

**Affiliations:** 1 School of Life Science and Technology, University of Electronic Science and Technology of China, Chengdu 611731, China; zmh@std.uestc.edu.cn; 2 Defense Innovation Institute, Academy of Military Sciences (AMS), Beijing 100071, China; 3 School of Information and Communication Engineering, University of Electronic Science and Technology of China, Chengdu 611731, China; bocheng_qian@std.uestc.edu.cn; 4 Academy of Medical Engineering and Translational Medicine, Tianjin University, Tianjin 300072, China

**Keywords:** EEG, dry electrodes, BCI, emotion recognition, fatigue detection, motor imagery, steady-state visual evoked potentials, artificial intelligence

## Abstract

As brain–computer interface (BCI) technology continues to advance, research on human brain function has gradually transitioned from theoretical investigation to practical engineering applications. To support EEG signal acquisition in a variety of real-world scenarios, BCI electrode systems must demonstrate a balanced combination of electrical performance, wearing comfort, and portability. Dry electrodes have emerged as a promising alternative for EEG acquisition due to their ability to operate without conductive gel or complex skin preparation. This paper reviews the latest progress in dry electrode EEG systems, summarizing key achievements in hardware design with a focus on structural innovation and material development. It also examines application advances in several representative BCI domains, including emotion recognition, fatigue and drowsiness detection, motor imagery, and steady-state visual evoked potentials, while analyzing system-level performance. Finally, the paper critically assesses existing challenges and identifies critical future research priorities. Key recommendations include developing a standardized evaluation framework to bolster research reliability, enhancing generalization performance, and fostering coordinated hardware-algorithm optimization. These steps are crucial for advancing the practical implementation of these technologies across diverse scenarios. With this survey, we aim to offer a comprehensive reference and roadmap for researchers engaged in the development and implementation of next-generation dry electrode EEG-based BCI systems.

## 1. Introduction

As a non-invasive technique for recording brain activity, electroencephalography (EEG) plays a crucial role in decoding neural signals and translating them into actionable commands, owing to its objectivity, reliability, and high temporal resolution. Simultaneously, the growing availability of computing resources and the rapid progress of artificial intelligence (AI), particularly in the field of machine learning, has significantly expanded the impact of EEG-based applications, thereby accelerating the development of brain–computer interface (BCI) technology [1,2,3]. These advancements have driven substantial progress across diverse domains, including text input [4,5,6], prosthetic control [7,8,9], neurorehabilitation [10], and affective computing, such as emotion recognition [11,12,13].

As the primary transducer for effective EEG signal acquisition, EEG electrodes serve as the front-end interface for signal amplification and processing. They represent one of the key technologies that must be optimized to promote the practical implementation of BCI systems. Wet electrodes are widely regarded as the gold standard in current EEG acquisition technology [14,15], while they also present practical limitations. During extended recording sessions, the conductive gel may dry out and must be reapplied to maintain consistent low impedance [15,16]. After data collection, residual gel on the scalp and EEG hardware, including electrodes and electrode caps, requires thorough cleaning. These procedures are typically time-consuming and labor-intensive, which reduces the convenience, portability, and usability of EEG-based BCI systems in daily or mobile scenarios [16].

In response to these limitations, dry electrode EEG systems have received increasing attention as a practical and user-friendly alternative. Unlike wet electrodes, dry electrodes eliminate the need for conductive gel and offer several advantages, including quicker setup, greater reusability, and suitability for long-term or mobile EEG monitoring applications [17,18]. These attributes significantly enhance user comfort and improve the applicability of EEG systems in real-world scenarios, especially in wearable technologies and consumer-oriented applications.

The portability of dry electrodes often leads to a reduced signal-to-noise ratio. To achieve a balance between portability and signal quality for enhanced recognition performance, current research primarily advances along two paths. One approach focuses on optimizing hardware design to improve signal acquisition at its source. The other seeks to enhance signal quality and recognition performance through post-processing, utilizing AI techniques, including machine learning and deep learning, to improve classification accuracy and robustness via signal preprocessing, feature extraction, and pattern recognition algorithms. Existing reviews predominantly emphasize hardware design progress, covering structural choices, design optimization, signal quality, impedance characteristics, and manufacturing processes, as shown in Table 1. For instance, Lopez-Gordo et al. detailed the electrochemical properties and stability of dry electrodes in their 2014 publication [18]. Fu et al. systematically explored dry electrode development from sensing principles to device fabrication [19]. Shad et al. focused on analyzing noise sources and impedance within amplifier systems [15]. Yuan et al. expanded their 2021 review to include non-invasive electrodes, including wet and semi-dry electrodes [20].

Distinct from previous reviews, this paper provides a comprehensive overview of the latest advancements in dry electrode EEG hardware technology and systematically assesses progress in specific BCI application areas from the perspective of signal post-processing. It addresses the gap in system-level performance evaluations within application scenarios that is often overlooked in existing reviews. The targeted applications include emotion recognition, fatigue and drowsiness detection, motor imagery, and steady-state visual evoked potentials. In particular, we highlight the progress made by integrating the selection of data acquisition instruments, experimental paradigms, and the design of machine learning algorithm architectures. By summarizing recent advancements and identifying key challenges, we aim to bridge the gap between hardware innovation and real-world deployment. This application-focused perspective provides a more comprehensive understanding by connecting hardware advancements to system-level performance in practical BCI environments. The main contributions of this paper are as follows:

- We systematically investigate the latest advancements in dry EEG hardware, with a focus on its architectural innovations and material developments. This enables researchers to draw insights from alternative hardware designs, facilitating the optimization of dry electrode performance at the front end of signal extraction.
- We focus on organizing and presenting the latest research progress of dry electrode EEG systems across various BCI application fields, systematically arranging the existing studies in terms of both timeline and logical progression. This approach not only significantly enhances understanding of the current status and development trends for readers but also assists researchers in selecting appropriate algorithms based on specific situations and diverse needs.
- We critically discuss current challenges and unresolved issues within the BCI domain, offering insights and outlining promising future research directions to guide subsequent studies.

## 2. Classification of Dry Electrodes

The emergence of dry electrodes addresses the inconvenience associated with traditional wet electrodes. Instead of relying on hard-to-clean conductive gel as an ion-exchange medium, dry electrodes leverage their structural design to establish direct contact with the scalp. Based on their structural characteristics, dry electrodes can be categorized into the following types: microelectromechanical system (MEMS) dry electrodes, dry non-contact electrodes, and dry contact electrodes.

### 2.1. MEMS Dry Electrodes

**Figure 1:**
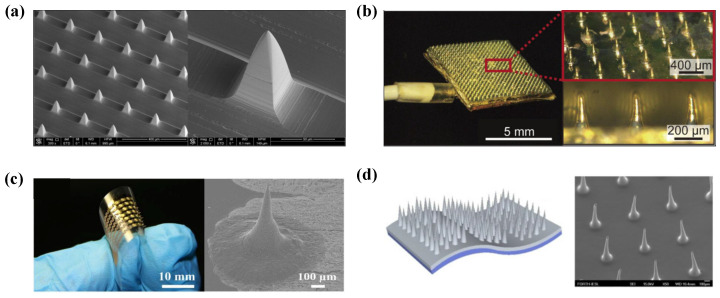
Examples of microneedle array dry electrodes. (**a**) Silicon microneedle-based dry electrode (reprinted with permission from [26], © 2013 Elsevier). (**b**) Polymer microneedle electrodes (reprinted with permission from [48], © The Japan Society of Applied Physics). (**c**) Flexible microneedle array electrode [49] (reprinted under CC-BY license). (**d**) SU-8 microneedle electrodes (reprinted with permission from [36], © 2016 Elsevier).

MEMS dry electrodes are a type of non-invasive EEG acquisition electrode developed using micro/nano-fabrication technology [26,27]. These electrodes utilize a microneedle array to gently penetrate the stratum corneum, which refers to the outermost layer of dead skin cells [28,29,30]. This design helps reduce the contact impedance between the skin and the electrode, thereby improving the quality of the recorded EEG signals [31]. Several examples of these electrodes are illustrated in Figure 1. Common materials used in the fabrication of microneedle array dry electrodes include silicon, metal, and polymers [32].

Silicon is among the first materials adopted for microneedles due to its mature microfabrication technology [33,34]. However, silicon-based microneedles necessitate stringent processing conditions, resulting in elevated manufacturing costs and low production efficiency. This renders them impractical for large-scale deployment. Furthermore, the inherent brittleness of silicon increases susceptibility to fracture under mechanical stress or suboptimal geometric design, posing injury risks during application. Silicon also exhibits significant biocompatibility limitations compared to alternative materials. Residual fragments embedded in tissue may cause severe complications, including potential life-threatening sequelae. Similar issues exist with other brittle materials, such as glass and ceramics, further limiting their clinical applicability in EEG acquisition.

Compared with brittle materials such as silicon, glass, and ceramics, polymer materials exhibit superior biocompatibility and mechanical flexibility. As a result, polymer microneedles are less likely to fracture abruptly during skin penetration, and their enhanced biocompatibility reduces the risk of adverse immune responses or tissue rejection. Commonly used polymers for fabricating these electrodes include polydimethylsiloxane (PDMS) [35], SU-8 [36], parylene C [37], polymethyl methacrylate [38], and polyimide [39]. To ensure electrical conductivity, the surface of polymer microneedles is typically coated with conductive metals such as gold (Au) [40], silver (Ag), platinum (Pt) [41], or nickel (Ni) through deposition or electroplating techniques. Despite these advantages, polymer microneedles still suffer from a critical limitation, as their inherently low hardness and stiffness often prevent them from effectively penetrating the stratum corneum. In addition, their lack of intrinsic conductivity requires extra fabrication steps to apply conductive coatings, which increases complexity and limits the broader application of polymer-based microneedle electrodes in practical EEG acquisition systems.

Metal materials have emerged as the preferred choice for fabricating microneedles or microneedle electrodes due to their excellent biocompatibility and high mechanical strength. Commonly used metals include titanium (Ti) [42,43] and stainless steel [44,45]. These materials help overcome the fragility and limited functionality associated with silicon and polymer-based microneedles. However, the fabrication of metal microneedle electrodes predominantly relies on processes such as ion etching and electrochemical corrosion, which remain complex and time-consuming. As a result, current methods still face challenges in achieving efficient and scalable mass production.

Currently, a widely adopted approach involves integrating a flexible substrate with a rigid microneedle array [30,41,46,47]. Flexible substrates such as PDMS and polyimide conform well to the curvature of the scalp, which helps reduce mechanical stress at the interface between the electrode and the skin and enhances user comfort. Meanwhile, the rigid microneedles possess sufficient mechanical strength to effectively penetrate the stratum corneum, thereby ensuring low impedance contact and maintaining high signal quality. However, compared to single-material and monolithically fabricated electrodes, this composite structure requires additional fabrication steps and more precise process control, resulting in higher material and manufacturing costs. Moreover, the insertion of microneedles into the skin may cause discomfort and carries a potential risk of infection. Examples of MEMS-based microneedle electrodes are illustrated in Figure 1.

### 2.2. Dry Non-Contact Electrodes

**Figure 2:**
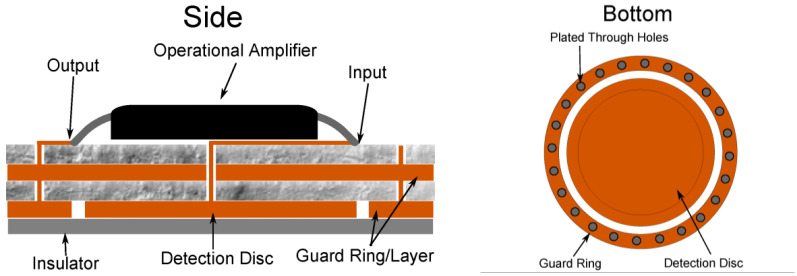
Example of dry non-contact electrodes [51], reprinted under CC-BY license.

Dry non-contact electrodes are distinguished by the absence of direct physical contact between the electrode and the skin. Instead, a dielectric layer such as a textured fabric, oxide, ceramic, plastic, or organic material is placed between the electrode and the skin surface [50]. EEG signals are acquired through the formation of a capacitive coupling circuit via this intermediate medium. This approach eliminates the need for direct current conduction on the skin, thereby enhancing both safety and comfort, as shown in Figure 2.

Chen et al. developed a non-contact dry electrode for EEG acquisition through the hair layer, featuring a copper disc electrode with a diameter of 20 mm and a thickness of 2 mm. A unity-gain operational amplifier ensures high input impedance, while a bandpass filter and adaptive mechanical structure are integrated to effectively suppress motion artifacts [52]. Lee et al. developed a dry EEG electrode utilizing a carbon nanotube (CNT) and adhesive PDMS composite for capacitive signal acquisition. The design featured hundreds of conductive pillars coated with a Parylene C insulating layer, integrated onto a conductive disk. A CNT/aPDMS layer was affixed to the disk to facilitate biosignal transmission to the pillars [53]. Experimental results showed the CNT/aPDMS electrode achieved an SNR of 3.71 ± 0.17 dB, outperforming a control electrode without CNTs (2.79 ± 0.13 dB). Additionally, motion-induced signal deviation was 1.4 ± 0.21 times higher than conventional wet electrodes, while the electrode without CNTs exhibited 5 ± 0.77 times higher deviation, highlighting CNT/aPDMS’s significant role in enhancing signal stability and motion resistance. Dabbaghian et al. introduced an eight-channel dry contactless EEG device weighing only 9.2 g. The complete circuit and active shielding were integrated onto a 4-layer flexible polyimide substrate, with a rechargeable battery and a 13 × 17 mm^2^ rigid circuit board connected at both ends of the main board [54].

In general, non-contact dry electrodes offer superior comfort and convenience, making them well-suited for wearable devices and everyday applications. However, they face several notable challenges. Without the aid of conductive gel, electrolytes, or other interface enhancers, the electrode–skin impedance can reach several megohms. Additionally, the acquired signals are more susceptible to motion artifacts. Although recent studies have made progress in addressing these issues [55,56], the signal quality of non-contact electrodes remains lower than that of contact or microneedle-based electrodes.

### 2.3. Dry Contact Electrodes

**Figure 3:**
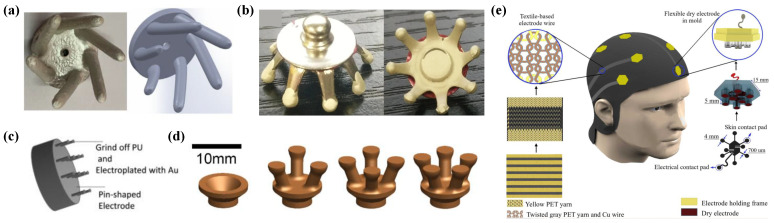
Examples of dry contact electrodes. (**a**) Claw-shaped electrodes [59] (reprinted under CC-BY license). (**b**) Claw-shaped electrodes [60] (reprinted under CC-BY license). (**c**) Pin-shaped electrodes (reprinted with permission from [63], © 2018 Elsevier). (**d**) Finger-shaped electrodes [62] (reprinted under CC-BY license). (**e**) The clutter-free e-textile EEG cap (reprinted with permission from [68], © 2025 Elsevier).

Dry contact electrodes refer to electrodes that obtain EEG signals through direct contact with the skin. While structurally similar to traditional wet electrodes, they eliminate the need for conductive gels, thereby reducing the risk of skin irritation or allergic reactions and enhancing user comfort and reusability. To address the challenges associated with signal acquisition quality, researchers have introduced a variety of structural innovations. Several examples of these electrodes are presented in Figure 3.

Currently, comb-shaped and claw-shaped designs are the two most commonly adopted electrode structures in both commercial products and laboratory research [57,58]. Recently, Wang et al. developed a claw-shaped dry electrode with a 20 mm base, a 2 mm central hole, and five 2 mm electrode claws evenly distributed along the edge. Coated with a TPU–Ag mixture (2:1 mass ratio), the electrode achieves an electrode–skin impedance of approximately 100 Ω [59]. Xing et al. developed a system designed for high-speed steady-state visual evoked potential applications. The electrode features a claw-shaped structure with a diameter of 14 mm and comprises eight protruding fingers, each measuring 6 mm in length and 2 mm in diameter. Constructed from thermoplastic polyurethane (TPU), the electrode is coated with Ag conductive ink to enhance electrical conductivity. Additionally, the tips are further treated with a mixed Ag/AgCl conductive ink to improve electrochemical performance [60]. Finger electrodes, a variant of comb-like or claw-like structures, have been introduced in recent years. For example, Tong et al. developed printed electrodes based on existing dry electrode designs [61]. The incorporation of a rounded finger-shaped structure facilitates penetration through hair while mitigating the discomfort typically associated with conventional sharp-needle electrodes [62].

To enlarge the contact interface between the electrode and the skin. Gao et al. proposed a flexible needle-shaped dry electrode in which the pins are made of carbon fiber, and the needle tips are further processed into carbon fiber bristles. This design effectively combines low impedance with enhanced softness and comfort [63]. Vasconcelos et al. proposed a dry arch electrode composed of five semicircular Ag/AgCl-coated arches arranged in parallel on a shared flexible TPU substrate. The TPU substrate was fabricated using additive manufacturing, ensuring both flexibility and structural integrity [64]. Fiedler et al. employed an improved version of their earlier design [65,66] by incorporating 30 chemically silver-plated needles, each with a spherical tip diameter of 1 mm, a height of 6 mm, and a center-to-center spacing of 2.4 mm. All needles were mounted on a shared base plate made of polyurethane [67].

Recently, textile electrodes have been extensively applied in the development of dry EEG electrodes. Owing to their softness, flexibility, and comfort, they are well-suited for monitoring and recording key electrophysiological signals, delivering functional electrical stimulation, and targeting specific muscle stimulation. Tseghai et al. proposed a washable and flexible textile-based dry EEG electrode fabricated from conductive cotton fabric coated with a PEDOT:PSS/PDMS conductive polymer composite, achieving a surface resistivity of 67.23 ΩΩsqsq [69]. López-Larraz et al. introduced the first garment capable of measuring brain activity, in which the electrodes, signal transmission pathways, and cap structure are entirely composed of wire, fabric, and smart textile materials, without relying on any metal or plastic components. The system incorporates four electrodes made from three-strand silver-coated nylon conductive yarn with a linear resistance of 114 ΩΩmm. The Fp1 and Fp2 electrodes cover an area of 2.8 cm^2^, while the F7 and F8 electrodes span 7.4 cm^2^ [70].

In addition to the aforementioned types, several advanced dry electrode devices have been developed. Li et al. proposed a flexible nanozyme/iridium-titanium dioxide ($\mathrm{SAN}/\mathrm{Ir}-\mathrm{Ti}O_{2}$) dry electrode for EEG signal acquisition. In this design, the SAN nanozyme helps maintain skin moisture balance, while the integration of iridium nanoparticles with titanium dioxide nanotube arrays facilitates efficient electron transfer. Consequently, the electrode achieved an average impedance of 19.9 kΩ in hairy regions [71].

While the above advances reflect the important contribution of custom-developed systems in research settings, it is equally important to investigate standardized solutions. Therefore, representative commercial dry electrode EEG devices with wider accessibility and potential for practical deployment are presented below. NeuroSky has launched a series of dry electrode EEG devices. The first-generation product MindSet features two electrodes, with one positioned on the user’s ear serving as a reference and the other placed on the forehead to capture EEG signals from the Fp1 site. Building upon this design, NeuroSky subsequently released MindWave, which is also a single-channel EEG device. It is more oriented toward educational and gaming applications, and its primary function is to monitor users’ attention and meditation levels. As a research-grade wireless dry electrode EEG acquisition system, the DSI-24 is developed by Wearable Sensing. It features 24 electrodes arranged according to the international 10–20 system and has demonstrated effectiveness in various fields, including neurofeedback, affective computing, and cognitive stress assessment. Another product, the Muse series, which is developed by the Canadian company InteraXon, offers consumer-grade EEG devices that utilize dry electrode technology. The first-generation Muse device, Muse 1, features four dry electrodes positioned at TP9, AF7, AF8, and TP10 for EEG signal recording. The second-generation Muse 2 builds on this design by incorporating flexible dry electrodes, improving comfort, and expanding its application from meditation feedback to sleep monitoring. Cognionics dry electrodes have proven to be invaluable in numerous fatigue detection studies [72,73,74,75,76]. Its core advantages include high channel density of up to 72 channels, integrated Flex Sensors’ flexible electrode technology and fully wireless design. High channel density enables more detailed and accurate signal acquisition. Flexible electrode technology ensures a more comfortable wearing experience and more stable skin contact, thereby improving signal quality and reducing the risk of discomfort during long-term use. In addition, the fully wireless design significantly improves the portability and ease of use of the system.

## 3. Key Applications

**Figure 4:**
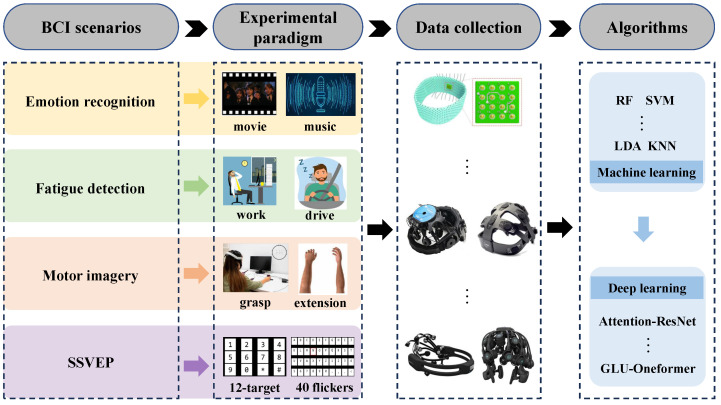
The key applications of BCI.

Over recent decades, research on human brain function has undergone a paradigm shift from theoretical exploration toward practical engineering applications. Advancements in AI have significantly empowered computational signal enhancement techniques for dry electrode electroencephalography, consequently accelerating the development of portable dry-EEG-based brain function recognition systems suitable for daily life implementation. This section will discuss and summarize the recent application progress of the dry electrode EEG system in the research fields of emotion recognition, fatigue and drowsiness detection, motor imagery (MI), and steady-state visual evoked potential (SSVEP), as shown in Figure 4.

This systematic review was conducted in accordance with the Preferred Reporting Items for Systematic Reviews and Meta-Analyses (PRISMA) guidelines [77]. A comprehensive literature search was carried out to identify recent studies on the architecture and application of portable dry electrode EEG in BCIs. The search covered the period from January 2019 to July 2025 and was conducted across four electronic databases: Google Scholar, Springer Link Online Libraries, Multidisciplinary Digital Publishing Institute (MDPI), and ScienceDirect (Elsevier). Basic search settings were used to identify topics relevant to our research interests. The search terms included dry electrode EEG, artificial intelligence, emotion recognition, fatigue and drowsiness detection, motor imagery, and SSVEP. The initial search was then refined to include studies published in the past six years. Further refinement was carried out by article type, excluding book chapters, conference abstracts, graduate theses, and review articles. The search was limited to publications in English. Ultimately, more than 20 articles on hardware and over 50 articles on major BCI application scenarios were identified.

The introduction follows a clear and consistent logical structure. It begins by outlining the importance and practical requirements of each application area, then proceeds to examine technical advancements related to dry electrodes. These advancements include both equipment optimization and developments in signal processing algorithms, with particular attention to the adoption of machine learning and deep learning methods. The narrative then moves on to present specific research findings, performance metrics, and progress in practical implementations. This structure reflects a comprehensive progression from technological adaptation to real-world application, with a focus on addressing the key challenges of dry electrode usage, particularly the need to balance signal quality, portability, and overall performance.

### 3.1. Emotion Recognition

Emotion serves as a vital mode of communication between individuals and the external world, reflecting physiological and psychological states that directly influence behavior, decision-making, and are closely linked to physical health [78]. In recent years, with the rapid advancement of intelligent human–computer interaction (HCI) technologies, EEG-based affective computing has garnered increasing attention [79]. Enabling machines to perceive and understand human emotions, and to respond in an intelligent and user-friendly manner, has become a prominent research focus for achieving more harmonious and user-friendly HCI [80,81]. However, traditional wet electrode EEG systems present limitations in terms of convenience and user experience, which have spurred research and development into portable dry electrode systems. This section will review current research on emotion recognition utilizing portable dry electrode EEG, as listed in Table 2.

Early studies primarily focused on verifying the feasibility of dry electrodes. For instance, one study compared the signal differences between wet and dry electrodes, highlighting that while dry electrodes are more susceptible to environmental noise, this issue can be effectively mitigated through the use of machine learning algorithms [82]. Specifically, Emsawas et al. employed the IMEC dry electrode system to extract features from eight-channel EEG signals, which were then processed using convolutional neural networks, specifically the deep convolutional networks (ConvNet) and shallow ConvNet models. These features were subsequently input into a long short-term memory (LSTM) network for emotion classification, achieving an accuracy of 71.7% in binary classification tasks [82].

Subsequent studies have specifically addressed the inherent limitations of dry electrodes, particularly in terms of material and structural design. For instance, copper-based silver sintering technology has been employed to enhance the contact area between the electrode and the skin, optimizing both stability and biocompatibility for long-term recordings. Demonstrating this approach, Rad et al. developed a dry EEG electrode using silver-copper sintering technology and collected EEG signals from 20 subjects listening to musical stimuli. By integrating type-2 fuzzy sets (FT2) with a deep convolutional graph network (DCGN) for pattern recognition on signals from the Fp1, Pz, and Fz channels, their system achieved an impressive 99% accuracy in a binary classification task [83]. For specific populations, such as the elderly, dry electrode devices with textile frames have been developed [57]. These devices feature a soft, lightweight design that reduces pressure during wear, while balancing signal quality and user comfort. Additionally, other studies have proposed miniaturized, low-cost homemade dry electrode devices to overcome the size and cost limitations of commercial equipment, making them more suitable for specific application scenarios. Mai et al. employed a self-developed wireless dry electrode device to collect eight-channel EEG signals from eight subjects while they watched emotional videos. Six entropy metrics were then input into a support vector machine (SVM), a multilayer perceptron (MLP), and a 1D convolutional neural network (CNN) for three-level emotion classification. The experimental results demonstrated that the accuracies for the subject-dependent and subject-independent strategies were 85.81% and 78.52% [84].

**Table 2:** Summary of the progress of emotion recognition based on dry electrode EEG.

Ref.	Year	Device	Channels	Model	Category	Performance
[85]	2019	Self-made	3	Model stacking (GBDT+RF+SVM)	Happy, fear, peace, disgust	subject-independent: 81.30%
[57]	2020	Self-made	4	CNN	Negative, positive	subject-dependent: 81.32%
[86]	2020	IMEC	8	RF	Valence, arousal	subject-dependent: Valence: 0.71 and arousal: 0.62
[82]	2020	IMEC	8	Deep ConvNet	Positive, negative	subject-dependent: 71.7 ± 15.5% (dry)
				+ LSTM		subject-dependent: 70.3 ± 22.5% (wet)
[84]	2021	Self-made	8	SVM/MLP/1D-CNN	Negative, neutral, positive	subject-dependent: 85.81% subject-independent: 78.52%
[87]	2021	Helmate8	8	KNN/SVM/ANN/ LDA/RF/LR	Valence	subject-dependent: 96.1%
[88]	2022	Muse2016	4	KNN/SVM/NN/ NB/RF/GBM	Happy, scared, calm, and bored	subject-independent: 85.01%
[89]	2023	Helmate8	8	SVM/KNN/ANN	Valence, arousal	Valence: 76.76 ± 3.83% Arousal: 77.86 ± 7.88%
[90]	2023	DSI-24	24	CD-EmotionNet	Disgust, fear, anger, sad, neutral, amusement, tenderness, and happiness	subject-dependent: 64.47 ± 10.17%
[83]	2024	Self-made	3	FT2 + DCGN	Negative, positive	subject-independent: 99%
[91]	2025	DSI-24	24	CDRC	Happy, fear, neutral, angry, and sad	subject-dependent: 60.05 ± 9.35% subject-independent: 58.18 ± 15.57%

From the perspective of signal processing and feature extraction, early studies relied on traditional signal processing methods to extract manual features. For example, the delta, theta, alpha, and other rhythmic bands of EEG signals were decomposed of wavelet transform and fast fourier transform (FFT), and the power spectral density (PSD) or differential entropy (DE) was calculated as emotion-related features [82,88]. Specifically, Sofian Suhaimi et al. used Interaxon Muse 2016 to collect four-channel EEG signals induced by VR stimulation materials, and achieved an accuracy of 85.01% for four classifications using the SVM classification model. In view of the nonlinear characteristics of the signal, entropy features such as permutation entropy (PEE) and sample entropy (SAE) are introduced to quantify the randomness of the signal to distinguish emotional states [84]. In addition, based on the theory of cerebral hemispheric asymmetry, spatial features are extracted by analyzing the signal differences across regions such as the frontal lobe, temporal lobe, and other areas. Apicella et al. used the Helmate8 eight-channel dry electrode system to collect EEG signals from 25 subjects and applied a variety of machine learning models for pattern recognition, with the shallow artificial neural network (ANN) achieving the highest average accuracy of 96.1% [87]. Amrani et al. also employed the Helmate8 to collect EEG signals from 26 subjects while they viewed emotional pictures. Three commonly used machine learning models, including K-nearest neighbor (KNN), SVM, and ANN, were adopted to classify valence and arousal. To achieve personalization, the researchers employed an incremental learning method, gradually incorporating high-quality data, with a standard deviation of 6.76 ± 3.83%, while for arousal it was 77.86 ± 7.88% [89].

With the advancement of deep learning, research has increasingly focused on automatic feature learning. CNNs, particularly 1D-CNN and shallow CNN, have become widely used for extracting spatiotemporal spectral features from EEG signals. Their hierarchical structure allows them to automatically capture both local patterns and global relationships within the data [82,84]. Recurrent neural networks are highly effective at processing the temporal characteristics of EEG data. When combined with CNNs, LSTMs can simultaneously model both spatial and temporal information, enhancing the network’s ability to capture complex patterns in the EEG signals [82]. In response to the “information sparsity” of few-channel signals, models based on graph convolutional neural networks (GCNs) create brain region connection maps to effectively capture the spatial correlations between the limited number of channels. This approach enhances the model’s ability to leverage the available data by focusing on the relationships between different brain regions [83,90]. Liu et al. collected EEG signals from 25 subjects using the DSI-24 device. They employed a novel cross-device EEG transfer network, CD-EmotionNet, which incorporates feature aggregation for eight-category emotion recognition. The model consists of a base emotion model designed to extract spatial-spectral-temporal EEG features and perform emotion classification, coupled with a cross-device transfer learning strategy. This approach achieved a classification accuracy of 74.47 ± 10.17% [90].

It is worth noting that deep learning models have significantly enhanced performance in complex scenarios. When combined with transfer learning, deep ConvNet can effectively address the issue of insufficient dry electrode data and noise. By transferring model parameters trained on wet electrode data to dry electrode scenarios, this approach improves the reliability and accuracy of emotion recognition using dry electrodes [82]. Meta-learning frameworks use global information from full-channel EEG to assist in feature learning of few-channel signals through cross-device knowledge transfer, and have verified their effectiveness on the self-built CDEED dataset and public datasets such as SEED and DEAP [90]. Self-supervised learning aligns the feature representations of dry and wet electrode signals to reduce dependence on labels, and has achieved performance that exceeds that of supervised methods on low-quality dry electrode signals. Self-supervised learning aligns the feature representations of dry and wet electrode signals to reduce dependence on labels, and has achieved performance that exceeds that of supervised methods on low-quality dry electrode signals [91].

Looking back at the progress in recent years, it is evident that research on emotion recognition based on dry electrode EEG has transitioned from the technical verification stage to practical exploration. The core advancements are reflected in the successful balance achieved between signal quality and wearable experience through innovations in material and structural design. Additionally, feature extraction and model design have shifted from being “manually dependent” to being “data-driven,” significantly enhancing robustness in complex scenarios. Personalization and cross-scenario adaptation have become key factors in addressing individual differences and overcoming the limitations of few-channel systems. In the future, as hardware technologies, algorithm models, and standardization systems continue to improve, dry electrode EEG emotion recognition is poised to achieve large-scale applications in fields such as mental health monitoring and intelligent HCI.

### 3.2. Fatigue and Drowsiness Detection

Fatigue and drowsiness are significant risk factors that can seriously compromise cognitive function, reaction time, and decision-making, particularly in safety-critical situations such as driving. Consequently, accurate and timely detection of fatigue and drowsiness is essential for accident prevention and public safety [92]. In recent years, dry electrode EEG devices have been increasingly adopted due to their comfort, ease of use, portability, and suitability for long-term, real-world monitoring. In this context, a growing body of research has demonstrated the effectiveness of dry electrode EEG systems in real-time monitoring of fatigue and drowsiness, as listed in Table 3.

For drowsiness detection, traditional machine learning methods typically rely on manual feature extraction, such as the spectral power ratio of EEG signals. In contrast, deep learning techniques, including CNN and residual network (ResNet), can automatically learn relevant features from the raw data. For example, Zhu et al. utilized CNN with the inception module to process EEG signals [76]. Building on their prior work, the team progressively refined their EEG-based drowsiness detection framework by transitioning from high-accuracy CNNs to attention-based ResNet models. This enhancement allowed for improved feature prioritization and greater real-world viability, achieving an accuracy of 93.35% [94]. Furthermore, feature selection has emerged as a critical research area. Sagila et al. employed a random forest algorithm to select 11 optimal features from a set of 52 EEG features [97], while Arif et al. compared various feature selection methods, concluding that combining spectral features with time correlation yields the best classification performance, effectively reducing computational costs [99].

To promote practical applications, research has focused on reducing the number of electrodes and the burden of wearing EEG devices. For instance, Sagila et al. used the consumer-grade Muse-2 headband, which has a limited number of electrodes, yet achieves high-precision detection through effective feature selection [97]. Arif et al. introduced a channel selection strategy, demonstrating that electrodes placed in specific brain regions, such as the prefrontal cortex (Fp1, Fp2) and the central area (C3, C4), can effectively reflect the state of drowsiness without the need for full brain coverage [99]. Additionally, reference [100] innovatively employed behind-the-ear EEG electrodes to minimize hair interference and enhance wearing comfort. This study also integrated TinyML technology, enabling real-time processing on edge devices, thus reducing data transmission energy consumption and minimizing latency.

Driving fatigue can impair the control and judgment abilities of drivers, becoming one of the leading causes of fatal traffic accidents. As a result, measuring driving fatigue has gained increasing attention in recent years. Building on the foundational research of EEG-based functional connectivity in driving fatigue detection, Wang et al. developed a phase synchronization framework to quantify the reorganization of brain networks across theta, alpha, and beta frequency bands. They utilized hand-crafted graph theory features, such as characteristic path length and clustering coefficient, along with key connections identified through the phase lag index (PLI), achieving a high classification accuracy of 96.76% in the beta frequency band using SVM [72]. To address the limitations of manual feature engineering and the static connectivity assumptions in traditional methods, the team subsequently developed an end-to-end model named AMCNN-DGCN [73]. This model integrates attention-based adaptive temporal filtering with multi-scale convolutional neural networks and DGCNs, enabling direct learning of spatial connectivity patterns from raw EEG data without requiring a predefined adjacency matrix such as PLI. Although the classification accuracy was slightly lower at 95.65%, it offers fully automated spatiotemporal feature learning and enhances the overall practicality of the system. Building on this foundation, the team proposed a self-attention channel connection capsule network (SACC-CapsNet), which integrates three core modules: a temporal channel attention mechanism, a channel connection attention module, and a capsule network with dynamic routing. SACC-CapsNet surpassed state-of-the-art models such as EEGNet and AMCNN-DGCN by 4.62 to 5.65% in terms of accuracy, sensitivity, specificity, and F1 score. Its capsule-based reconstruction technique preserves critical connectivity patterns, further improving model performance [75]. In another study, Xu et al. developed an EEG-based driving fatigue detection system for autonomous driving, distinguishing it from other methods by providing simultaneous identity authentication. The system utilizes a CNN-attention hybrid neural network architecture, enabling both individual identity authentication (PI) and driving fatigue state detection. In a 90-min simulated driving experiment with 31 subjects, the system achieved an identity authentication accuracy of 98.50% and a fatigue detection accuracy of 97.80% [95].

To promote a balance between performance and practicality, Sheykhivand et al. addressed the issue of data redundancy by combining compressed sensing (CS) with deep neural networks (DNNs) for the first time. This approach reduced both the storage and computational burden by compressing the EEG data while retaining essential information [98]. The results demonstrated an accuracy of 95% at a compression rate of 40% and 92% at a compression rate of 90%. The method achieved an accuracy of up to 99.1% without compression, highlighting its strong noise resistance and supporting efficient, real-time fatigue detection with minimal channels. From the perspective of optimizing model complexity, Sun et al. proposed an ultra-lightweight CNN, named SGL-Net, to reduce computational costs by simplifying the network structure, making it suitable for embedding on edge devices. Using a self-developed forehead dual-channel dry electrode EEG acquisition device, they combined the spectral group-guided lightweight CNN to develop an ultra-lightweight fatigue detection system. This system efficiently detected fatigue while minimizing computational resources, making it ideal for real-time applications on edge devices [102]. Extensive EEG signal features derived from multiple channels and frequency bands hold significant potential for fatigue detection. However, the inclusion of numerous features increases computational complexity, elevates the risk of overfitting, and reduces model interpretability. To address these challenges, researchers have developed optimized approaches. Notably, Liu et al. implemented a practical driving fatigue detection system utilizing only three frontal hairless EEG channels on the Mindo-4 Jellyfish platform. Their method extracted hybrid features from channel pairs, encompassing PSD, functional connectivity, and entropy metrics. By combining these EEG features with behavioral reaction time and employing data-driven feature selection for personalization, the system achieved robust fatigue state recognition. Utilizing a BP-AdaBoost classifier [96], it attained a single-subject accuracy of 92.7 ± 0.92% and a cross-subject accuracy of 77.13 ± 0.85%. In contrast, while also focusing on a minimal channel configuration, Wang et al. proposed an enhanced Transformer architecture named GLU-Oneformer [74]. This model integrated traditional EEG features with novel brain lateralization features, specifically asymmetry scores of power ratio and approximate entropy. To enhance feature discriminability, gated linear units (GLUs) were incorporated into both the attention and feedforward network modules of the Transformer. Furthermore, optimizing feature selection from a spectral perspective, Arif et al. employed Z-score normalization to fuse results from three feature selection techniques (MRMR, chi-square test, Relief F). This process identified delta, theta, alpha, and beta rhythm power as key spectral features. Utilizing a bagged decision tree ensemble classifier with data from a single right-frontal (F8) electrode, their optimized approach achieved a classification accuracy of 85.6%, recall of 89.7%, and F1-score of 87.6%. Critically, the execution time was minimized to merely 76 milliseconds, significantly reducing computational burden and hardware requirements [99].

Subsequent studies have progressively overcome scene limitations and focused on addressing the generalization problem. Luo et al. introduced a cross-scene domain adaptation method (CS2DA) to tackle the “domain offset” issue between laboratory data and real-world driving scenes. By integrating multi-source data (laboratory and real-world), they developed a generalization model that enhances detection accuracy in real environments. Additionally, they created two datasets: SEED-VLA for the laboratory scene and SEED-VRW for real driving scenarios. Specifically, this method addressed individual differences among subjects through adversarial learning and employs multi-kernel maximum mean discrepancy combined with conditional MK-MMD constraints to minimize scene-related discrepancies [101]. In terms of expanding application scenarios, Wang et al. extended EEG-based fatigue detection from the driving domain to agriculture, specifically focusing on beekeeping. They designed a 3D-CNN model tailored to the physical and mental fatigue characteristics of beekeepers, thereby filling a gap in fatigue detection for niche industries. This expansion also included the incorporation of various fatigue levels, further broadening the applicability of fatigue detection systems in unconventional settings [103].

In summary, existing research has progressively advanced from basic methods to more complex scenarios through three major directions: lightweight wearable devices, algorithm efficiency, and edge deployment. These advancements have systematically addressed the challenges of invasiveness, environmental sensitivity, and computing costs that are inherent in traditional methods, making emotion recognition and fatigue detection more practical and applicable across various domains.

### 3.3. Motor Imagery

MI involves subjects imagining the movement of body parts without executing actual physical movements, which activates key regions of the brain. These imagined movements stimulate a large number of neurons, leading to interactions between them. The electrical signals generated by these neuronal activations can be captured by EEG devices. MI-based EEG signals can control external devices such as wheelchairs or computer cursors, making them crucial for the development of assistive technologies that enable people with disabilities to interact and communicate with their environment. To further enhance the design of personalized assistive devices and ensure they are easy to use and comfortable for long-term wear, many studies have shifted from using traditional wet electrode systems to dry electrode systems. This transition is driven by the need for portability, convenience, and comfort in real-world applications. This subsection will review the recent advancements in dry electrode technology for MI applications, as listed in Table 4.

Early studies primarily focused on addressing the fundamental processing challenges associated with dry electrode signals, relying on traditional feature extraction and classification algorithms. Common spatial pattern (CSP) and its variants, such as filter bank CSP (FBCSP) and discriminative FBCSP (DFBCSP), are widely used for traditional feature extraction [104,105,106]. These methods work by extracting spatial features through the maximization of inter-class variance [107,108,109]. For instance, Olivas-Padilla highlighted that FBCSP enhances performance by applying CSP across different frequency bands, while DFBCSP further refines frequency band selection to better accommodate individual differences. Classification was performed using a modular network composed of four expert CNNs. The method was evaluated on a dataset recorded from eight subjects using an eight-channel OpenBCI device, achieving a maximum average accuracy of 76.62% [107]. For classification algorithms, shallow models such as SVM and linear discriminant analysis (LDA) are commonly employed [108,110]. However, due to the limitations of manual feature design, the accuracy in multi-class tasks often falls below 80% [107]. Casso et al. recruited 10 participants to perform left and right hand motor imagery tasks, with EEG signals recorded using a 32-channel Enobio system configured with seven different electrode settings (ranging from 8 to 32 channels). The experimental results showed that the Riemannian cut space (RTS) integrated with SVM achieved peak performance of 70% with 20 channels [110].

With the development of deep learning, the recent literature has started to utilize models such as CNN and LSTM to automatically extract spatiotemporal features directly from the original EEG signals, thereby overcoming the performance limitations of traditional methods. Spatiotemporal or time-frequency features of EEG are captured using 1D/2D convolution techniques [107,109,111]. For example, reference [107] designed both a single CNN and a multi-expert CNN, combined with DFBCSP to optimize the frequency band, significantly improving the classification performance for six different tasks (left and right hands, left and right feet, tongue, and idle state). Shajil et al. applied a single convolutional layer CNN to a CSP-filtered spectrum map, achieving two- and four-class MI classification. The experimental results showed that the method achieved an average accuracy of 95.18 ± 2.51% for binary classification and 87.37 ± 1.68% for four-class classification [109]. By combining CNN with LSTM/Transformer hybrid models, recent studies have addressed both spatial features and long-term temporal dependencies [111,112]. For example, Intarasopa et al. employed a CNN-LSTM hybrid model to achieve real-time classification of four types of motor imagery (left and right hands, feet, and idle state) using eight-channel dry electrode EEG [111]. Zare et al. introduced a Transformer-based model to tackle the data imbalance problem, thereby enhancing the robustness of MI intention detection [112].

**Table 4:** Summary of the progress of MI based on dry electrode EEG.

Ref.	Year	Device	Channels	Model	Category	Performance
[113]	2019	Emotiv Epoc	14	SBCSP-SBFS+NBPW	3	subject-dependent: 60.61%
[107]	2019	OpenBCI	8	DFBCSP+4 CNNs	5	subject-dependent: 76.62%
[114]	2020	EEG-HeroTM	11	sLDA	3	subject-dependent: 56.4 ± 8% (dry) subject-dependent: 62.3 ± 8.2% (wet)
[115]	2020	Muse	4	CNN+LSTM	2	subject-dependent: 96.50%
[109]	2020	Enobio	16	CNN	2 & 4	subject-dependent: 87.37 ± 1.68% subject-dependent: 81.90 ± 4.45%
[116]	2021	ActiCap Xpress Twist	31	MD-CNN	3	subject-independent: 58.44% (dry) subject-independent: 58.66% (wet)
[110]	2021	Enobio	32	RTS+SVM	2	subject-dependent: 70%
[117]	2022	CGX system	30	2-Conv-FBCNet	2	Healthy: 61.0% Stroke: 59.4%
[108]	2023	Self-made	3	LGBM+MVO	2	subject-dependent: 90.37%
[118]	2023	OpenBCI	14	SVM	2	73.50%
[111]	2024	g.tec Unicorn Hybrid Black	8	CNN+LSTM	4	Offline: 40.9 ± 16.9% Online: 35.9 ± 10.4%
[119]	2024	CGX system	32	SOTL	4	subject-independent: Stroke: 51.2 ± 0.17%
[120]	2025	CGX system	32	TSDA	4	subject-dependent: Stroke: 51.7%
[112]	2025	OpenBCI	16	Transformer	2	subject-dependent: 85.3%
[121]	2025	BlueBCI	8	Transfer learning-assisted GCN	2	Cross-validation: 71.19% Cross-session: 69.03%

To address the challenges of dry electrode signal noise and poor generalization across subjects and devices, the recent literature has introduced more advanced optimization techniques. These methods involve transferring knowledge from the source domain (such as wet electrode or healthy subject data) to the target domain (such as dry electrode or patient data), effectively compensating for small sample sizes or signal quality deficiencies [119,120,121]. For example, Ma et al. proposed a source model optimization transfer learning (SOTL) strategy, which transfers a model trained on healthy subject data to stroke patients, improving multi-task classification accuracy for unilateral upper limbs. Zhang et al. employed knowledge distillation and fine-tuning to transfer a model trained on 62-channel wet electrode data to 8-channel dry electrodes, addressing the problem of channel differences [121]. The method achieved a cross-validation accuracy of 71.19% and a cross-session accuracy of 69.03% on the eight-channel dry electrode EEG dataset. Additionally, some studies have focused on improving performance by optimizing model hyperparameters, such as the number of convolutional layers and learning rate [107,109]. For example, Ma et al. designed a spatiotemporal domain adaptation framework (TSDA) that optimizes domain distance using spatiotemporal mask kernel functions [119].

Recent studies have demonstrated that the application of MI has progressively shifted from basic classification tasks to real-world implementation in practical scenarios. Some studies focus on the recovery of motor function in stroke patients, designing MI tasks that better align with rehabilitation needs, such as “reaching” and “twisting” of unilateral upper limbs, and enabling active rehabilitation through BCI-driven devices like soft gloves [112,117,119]. For example, Zare et al. recruited three healthy participants to control a soft robotic glove for hand rehabilitation using MI EEG signals. Specifically, EEG data was acquired through a 16-channel dry electrode cap while the participants performed a fist-clenching/resting task. A pneumatic soft glove was controlled using the decoded MI intentions using a Transformer-based deep learning model. The system achieved a peak accuracy of 85.3% and an average AUC of 0.88 [112]. Some studies are also focused on the development of portable devices for long-term monitoring, by integrating dry electrode MI-BCI with wearable devices such as the Muse headband (four channels) and OpenBCI (eight channels) to enable home monitoring or daily control [110,111,115]. For instance, Garcia-Moreno et al. explored the application of the Muse headband in healthy aging, utilizing deep learning classification based on CNN and LSTM of left and right hand MI to support independent living for the elderly, and the verification accuracy was as high as 96.50% [115]. To better align with real-life applications, multi-class tasks and complex scenarios have also become key research areas. These have evolved from the initial two-class classification (such as left and right hands) to more complex multi-class tasks (including limbs, tongue, and idle states), and even “motor imagery + actual action” mixed scenarios, which significantly enhance the practicality of BCI systems [107,111,114]. For instance, Schwarz et al. recorded “reaching-grasping” actions of 45 subjects using three different EEG devices, including wet electrodes, water-based electrodes, and dry electrodes, to verify the feasibility of dry electrodes in decoding complex movements. In total, 80 participants executed hand-reaching and grasping movements, including a palm grasp of a glass and a lateral grasp of a spoon. By extracting movement-related cortical potential and low-frequency time-domain features, and applying the sLDA method, the decoding accuracy of the 11-channel dry electrode system was 56.4 ± 8% [114].

Based on the above research, we observe that current studies are focused on improving the performance and practical application of dry electrode MI-BCI systems. These studies demonstrate a technical progression, moving from basic feature extraction to advanced deep learning models and optimization strategies. Additionally, there has been an expansion in applications, ranging from laboratory-based classification tasks to rehabilitation therapy and portable devices. Collectively, these advancements establish a comprehensive technology chain for dry electrode MI-BCI, spanning signal processing to real-world implementation. This progression provides a multi-dimensional solution to address the limitations of dry electrode signals, promoting their integration into both clinical and everyday scenarios.

### 3.4. Steady-State Visual Evoked Potential

SSVEPs induced by flickering visual stimulation have been shown to be an effective method for controlling BCIs due to their high accuracy and fast response time. Recent advances in dry electrode EEG systems offer a promising solution for alternative and augmented communications in practical applications by providing a more comfortable, user-friendly, and portable alternative. This section explores the application of dry electrode EEG systems in SSVEP-based BCIs, focusing on their potential for improved performance and portability in various tasks, as shown in Table 5.

Existing research initially focuses on the material and structural innovation of dry electrodes to address the fundamental trade-off between signal quality and comfort. For the first time, a systematic classification of dry electrode types, such as microneedles and spring needles, was conducted, highlighting their limited application and low information transfer rate (ITR) in BCIs [60], thereby clarifying the direction for future research. In response to the need for high-density signal acquisition, a high-resolution dry electrode array design was proposed to overcome short-circuit issues that arise when the distance between wet electrodes or semi-dry electrodes is too small [122]. Furthermore, an innovative material design involving ($\mathrm{SAN}/\mathrm{Ir}-\mathrm{Ti}O_{2}$) dry electrodes has been introduced, which reduces contact impedance and enhances biocompatibility through a nanozyme biofilm [71]. This design was directly compared to wet electrodes to verify the improvement in signal quality.

**Table 5:** Summary of the progress of SSVEPs based on dry electrode EEG.

Ref.	Year	Device	Channels	Model	Category	Performance
[60]	2018	Self-made	8	TRCA	8	subject-independent: 93.2%, 92.35 bits/min
[122]	2019	Self-made	16	FBCCA	4	subject-dependent: 88.5%
[123]	2020	Self-made	1	IF-EMD	3	subject-independent: 90.7 ± 2.9%, 54.94 ± 5.41 bits/min
[124]	2020	Self-made	1	EA-SVM	4	subject-independent: 97.6%
[125]	2020	Self-made	1	Correlation+dual threshold	2	subject-independent: 83.5%, 39 bits/min
[126]	2021	NeuSenW	9	FBTRCA	12	subject-independent: 73.0 ± 3.0%, 201.2 ± 23.87 bits/min
[127]	2021	Self-made	8	ALPHA	12	subject-independent: 72.91 ± 4.87 bits/min
[128]	2023	Neuracle	24	FBCCA	60-character	subject-independent: 90.18%, 117.05 bits/min
[129]	2024	NeuSenW	9	VMD+WT+FBCCA	12	subject-independent: 72.46 ± 21.95%
[71]	2024	Self-made	8	MetaBCI	8	subject-independent: 92.8%
[130]	2024	OpenBCI	8	OACCA	40	subject-independent: 70.59 bits/min
[131]	2025	Avertus H10C	10	SEMSCS	8	subject-dependent: 87.5%, 346.8 bits/min

Secondly, to address the issue of dry electrode signal noise, significant breakthroughs have been made from the perspectives of signal noise reduction, feature extraction, and classification algorithms. To overcome the challenge of detecting high-frequency SSVEP (47/50/53 Hz), the iterative filtering empirical mode decomposition (IF-EMD) algorithm was proposed to resolve the mode aliasing problem of traditional EMD and enhance the high-frequency signal analysis capability [123]. In response to the noise characteristics of wearable devices, the noise reduction method was introduced, combined with the improved filter bank canonical correlation analysis (FBCCA) to boost feature extraction efficiency and improve the signal noise rate [129]. To address the issue of limited data from single-channel dry electrodes, SVM combined with evolutionary algorithms was employed to optimize parameters and enhance classification accuracy [124]. Furthermore, to compensate for the shortcomings of dry electrode performance, the align and pool for EEG headset domain adaptation (ALPHA) transfer learning framework was proposed, which utilizes wet electrode data to assist the dry electrode system and improves ITR through subspace alignment. Experimental results demonstrated that, when applied to a virtual dialing keyboard for the 12-target SSVEP-BCI speller task, ALPHA achieved an ITR of 72.91 ± 4.87 bits/min [127].

By combining technological breakthroughs with practical application scenarios, research has focused on addressing the “last mile” problem of dry electrode technology. A study focused on ADHD rehabilitation for children, integrating dry electrode SSVEP, AR glasses, and robot control to enhance rehabilitation engagement through gamified interaction while optimizing real-time delay and accuracy [127]. In the speller scenario, Zhang et al. designed the TSFC-SSVEP paradigm combined with an electrooculography (EOG) brain-controlled switch to reduce visual fatigue, enabling 60 command outputs and overcoming the limitation of a small number of commands in the dry electrode system [128]. Ma et al. addressed the integration challenge between wearable devices and external equipment by proposing a ROS-based framework (Gaitech BCI), enabling seamless control of dry electrode EEG and robotic devices [131]. The proposed squeeze-and-excitation attention module and multi-scale convolutions (SEMSCs) network achieved an ITR of 346.8 bits/min with 0.2 s data and 87.5% accuracy with 1 s data in within-subject experiments. Finally, focusing on neurorehabilitation scenarios, Li et al. developed a quick setup wearable SSVEP-BCI system and balancing system performance with user acceptance through training-free algorithms such as canonical correlation analysis (CCA) optimization [130].

To provide foundational resources for promoting technological iteration, Zhu et al. addressed the issue that existing public datasets lack large-scale wearable dry electrode SSVEP data [126]. A dataset containing 102 subjects, including comparisons between dry and wet electrodes, as well as long-term usage records, was constructed to fill the resource gap in practical research.

Based on the above literature, it can be observed that existing research centers on the practical application of dry electrode SSVEP-BCI, forming a progressive logical chain that spans from hardware (electrode design) to software (algorithm optimization), from technology (signal processing) to scenarios (rehabilitation, spelling, robot control), and from basic research to resource construction. This logical progression collectively drives the field forward, transitioning from laboratory verification to daily application.

## 4. Challenges and Future Opportunities

### 4.1. Challenges

For years, clinical and research-grade EEG systems have primarily relied on mature wet electrode technology, offering high-quality data suitable for a wide range of application scenarios and pattern recognition models. However, as BCI technologies are increasingly integrated into everyday life, there is a growing demand for systems that emphasize practicality, convenience, universality, and aesthetics. The development of high-performance electrodes and headsets that are lightweight, visually appealing, and easy to wear and operate has become an inevitable trend for scalp EEG acquisition. In response to these needs, dry electrodes have emerged as a promising alternative and have attracted significant attention from the research and industrial communities. However, critical questions remain regarding whether dry electrodes can deliver signal quality comparable to that of wet electrodes, as well as their optimal application domains and limitations. These issues warrant further systematic investigation in future studies.

#### 4.1.1. Hardware Design

While dry electrodes have addressed several limitations of traditional scalp wet electrodes, their performance, as reviewed in this study, remains highly dependent on the context in which they are used. Although some studies suggest that dry electrodes can achieve recognition results comparable to wet electrodes [114,116,118], these outcomes are typically observed under specific experimental conditions or within carefully controlled application scenarios. This dependence stems from three fundamental limitations inherent in current dry electrode architectures: signal attenuation due to high electrode–skin impedance, high sensitivity to motion artifacts, and poor individual adaptability.

Specifically, the lack of conductive gel makes it difficult for dry electrodes to effectively penetrate the stratum corneum and hair barrier, resulting in an impedance (>100 kΩ) much higher than that of wet electrodes (<10 kΩ) [15,18]. This high impedance directly causes significant attenuation of low-frequency signals (especially in the delta/theta band <4 Hz) [84], seriously affecting applications that rely on such signals, such as the recognition of emotions and fatigue states. Dry electrode structures are generally rigid and lack the damping effect of the gel layer. This leads to a high risk of mechanical decoupling between the electrode and the scalp during movement, resulting in unstable contact [54,56]. The direct consequence is an increase in baseline drift and a significant amplification of motion artifacts, which is further manifested as an increase of 30–40% in 50/60 Hz power line noise in dynamic scenes [60]. In addition, differences in hair density, scalp oiliness, and skull curvature between individuals make it difficult for dry electrodes to ensure consistent contact quality [58,69]. This contact instability is directly reflected in the recognition performance, as the accuracy of subject-independent verification is generally 10–15% lower than the subject-dependent results [90].

In summary, while dry electrode systems have shown promise in certain instances, they have yet to demonstrate consistent performance parity with conventional wet electrodes across a wide range of real-world settings. This inconsistency highlights the need for continued improvement in dry electrode technology to match the robustness and versatility of wet electrodes.

#### 4.1.2. Application Implementation

- Device fragmentation: The use of different EEG devices within the same application scenario leads to incomparable signal quality. For instance, commercial devices like DSI-24 [90,101] and consumer-grade devices such as Muse [88] exhibit significant differences in signal-to-noise ratio and impedance characteristics. Additionally, inconsistent channel configurations result in varying spatial coverage and feature extraction capabilities. Consequently, it becomes difficult to evaluate results across studies on a comparable basis. For example, in SSVEP-based applications, the ITR is influenced by both stimulation frequency and the number of channels, making it impossible to directly compare data across different studies.
- Paradigm fragmentation: Stimulation paradigms are not unified across studies, for instance, emotion induction may use VR [85,88], movies [84], music [83], or other methods. In MI studies, some classifications are based on hand movement tasks [113,118], while others involve imagined behaviors such as sitting or gait [116]. These variations in task design may introduce additional variables, further complicating the comparison of results across studies. Moreover, differences in subject groups further complicate comparisons, as some studies focus on healthy adults [107,108], whereas others involve patients [117,120], such as those undergoing stroke rehabilitation.
- Algorithm fragmentation: Within the same application context, numerous algorithm variants (over ten identified) exist, yet insufficient evidence currently supports definitive identification of the optimal machine learning approach. Furthermore, algorithm performance exhibits a strong dependence on hardware configuration due to the tight coupling between specific devices and application scenarios. Key factors include the number of channels and electrode placement. For example, in emotion recognition, a complex model like CD-EmotionNet combined with a 24-channel DSI-24 device achieved an accuracy of only 74.47% [90], while a simpler approach using the 4-channel Muse device and SVM reached 85.01% [88]. In SSVEP tasks, the ITR of a single-channel Oz electrode using IF-EMD differs by a factor of six compared to a multi-channel system using FBTRCA [123,126]. A significant computational efficiency-practicality trade-off is also evident. High-performance models such as SACC-CapsNet [75] and 3DCNN [76] impose substantial computational demands, hindering deployment on embedded platforms. In contrast, lightweight solutions like BP-AdaBoost [96] and decision trees [93,99] satisfy real-time constraints but deliver limited accuracy, typically ranging between 77% and 85%.
- Limited Generalization and Practical Applicability: The majority of current research primarily focuses on data analysis within subject-dependent scenarios in experimental design [87,101]. However, EEG patterns exhibit considerable variation between individuals, leading to a significant decline in the performance of existing models when applied to cross-subject scenarios. Furthermore, the majority of the results are still limited to offline processing [90,91,117], and substantial progress is required before these approaches can be effectively applied in real-world settings.

### 4.2. Future Opportunities

#### 4.2.1. Hardware Design

Given the current limitations, sustained innovation is crucial in several areas, including electrode materials, structural design, and ergonomic integration methodologies. Future research should prioritize the convergence of emerging material technologies and micro/nano-fabrication techniques with human-centered design principles [34], exploring materials with lower inherent noise, better biocompatibility, and more stable skin contact interfaces (such as nanocomposites, flexible conductive polymers), and designing electrode structures that can adapt to different head shapes, hair types, and even slight perspiration, thereby reducing noise introduction from the source. This approach will be essential for the development of portable scalp EEG electrodes that offer mechanical compatibility with skin tissue [132], enhanced user comfort [19], and electrical performance equivalent to wet electrodes. By focusing on these areas, it will be possible to create more effective and user-friendly dry electrode systems that can be applied across diverse environments and scenarios, expanding their practical use in both clinical and non-clinical settings.

#### 4.2.2. Application Implementation

- Establish standardized evaluation benchmarks: The diversity of equipment, experimental paradigms, and algorithmic approaches has resulted in significant fragmentation across existing studies, creating a high level of variability that hinders horizontal comparison and integration of research findings [22]. This fragmentation poses a challenge to the accumulation of shared knowledge and impedes the coordinated advancement of technology within the field. To address this issue, it is essential to establish and adopt standardized procedures for the use of dry electrode EEG systems in task recognition research. Future studies should prioritize collaborative efforts within consistent application scenarios, employing standardized stimulation materials, harmonized data acquisition protocols, and unified performance evaluation metrics, as has been successfully implemented in other research domains [133,134].
- Robust and adaptive signal preprocessing: Future research should explore advanced methods suitable for addressing the highly non-stationary and nonlinear nature of dry electrode signals, such as improvements to empirical mode decomposition and its variants, time-varying autoregressive models, and recursive quantitative analysis [123,126]. These techniques will enable more effective extraction of transient features and improved handling of signal disruptions. Furthermore, a systematic investigation into the integration of additional physiological signals, such as electrooculography, electromyography, electrocardiography, and electrodermal activity, is essential to enhance the identification and removal of specific artifacts, including blinking, muscle activity, and heartbeats, in dry electrode EEG systems.
- Algorithm–hardware co-design: Future research should promote lightweight architectures that address the requirements of embedded deployment, focusing on data compression, feature engineering, and computational efficiency of recognition models. By optimizing both algorithms and hardware in tandem, it will be possible to achieve high performance while ensuring real-time processing and practical usability.
- Enhance generalizability: Future research can leverage machine learning approaches such as transfer learning [135,136], contrastive learning [137], domain adaptation [138], and meta-learning [139] to address the key generalization challenges posed by subject-independent and dataset-independent variability. These advanced techniques offer promising solutions for enhancing the robustness and adaptability of dry electrode EEG systems across diverse user populations and application scenarios.
- Expanding Potential Clinical Applications: We have highlighted the use of motor imagery for helping stroke patients interact and communicate with their surroundings in our research. However, future research should explore broader clinical applications of dry electrode EEG systems. These include long-term home monitoring of motor fluctuations, such as tremor and dyskinesia, and the assessment of cognitive decline through quantitative EEG biomarkers. Additionally, dry electrodes could be employed for detecting ambulatory epileptic seizures and evaluating treatment responses in depression and anxiety disorders via frontal alpha wave asymmetry. By advancing these applications, dry electrodes have the potential to offer portable, real-time solutions for precise clinical assessment and personalized intervention, paving the way for improved remote healthcare options.

## 5. Conclusions

This comprehensive review outlines the substantial advancements in portable dry electrode EEG technology, particularly its growing role in practical BCI applications. The review highlights that innovations in dry electrode materials and designs, particularly in dry contact and dry non-contact configurations, have notably enhanced user comfort and practicality, positioning them as a viable alternative to traditional wet electrodes in numerous real-world settings. Noteworthy progress has been observed in key BCI paradigms, such as emotion recognition, fatigue detection, motor imagery, and SSVEP, with dry electrode EEG systems showing promising performance. These systems increasingly benefit from the integration of advanced signal processing and artificial intelligence algorithms, which help overcome inherent challenges related to signal quality. However, widespread adoption of dry electrode EEG technology remains hindered by several significant challenges. While the technology holds great promise for next-generation, user-centric BCIs, realizing its full potential will require continued efforts to address critical issues related to standardization and generalizability. By focusing on these pivotal areas, the field can accelerate the development of reliable, robust, and truly portable dry electrode EEG-BCI systems, paving the way for their integration into diverse clinical, assistive, and consumer applications.

## Figures and Tables

**Table 1:** Comparison of related surveys.

Ref.	Year	Details	Focus of Survey
[18]	2014	A review from the perspectives of material selection, structural design, and measurement performance, providing reference for evaluation in terms of electrochemical properties, stability, and related factors.	More focus is placed on hardware developments, which reviews progress up to 2014.
[21]	2019	A review of signal quality and usability in comprehensive comparison with wet electrodes.	More focus is placed on hardware advancements, which reviews three types of dry electrodes: gold-coated single pin, multiple pins, and solid gel electrodes.
[19]	2020	A review of the development of dry electrodes in terms of sensing principles, material selection, device fabrication, and measurement performance.	More focus is placed on the material characteristics of dry electrodes currently used for bioelectric signal monitoring, with a brief overview of their application scenarios.
[15]	2020	A review of impedance and noise in EEG systems, focusing on impedance types, dry electrode materials, noise sources, mitigation strategies, and amplifier–impedance interactions.	More focus is placed on recent hardware advancements, which reviews the latest amplifiers and electrodes from the perspective of impedance and noise.
[20]	2021	A review of the development of dry, wet, and semi-dry EEG electrodes in terms of material selection, structural design and performance evaluation standards.	More focus is given to the hardware development of non-invasive electrodes, not limited to dry electrodes.
[22]	2022	A review of consumer EEG devices and their effectiveness in emotion recognition reliability testing.	More focused on a single emotion recognition application scenario.
[23]	2022	A review of the fabrication of dry EEG electrode materials and the characteristics of the acquired EEG signals.	More focus is given to the hardware development.
[24]	2023	A review of the physical properties and signal performance of advanced EEG electrodes based on their skin-contact mechanisms.	More focused on hardware progress from the perspective of skin-contact mechanisms.
[25]	2025	A review of recent advances in the characterization, design, fabrication, and performance of carbon-based nanocomposites for EEG electrodes.	More focus is placed on the progress of carbon-based nanocomposite electrodes.
Ours	2025	A review of recent advances in the hardware design and application performance of dry electrode EEG systems across multiple typical BCI scenarios.	More focus is placed on the recent developments in dry electrode hardware design, and the research progress of dry electrode EEG systems in specific BCI application scenarios, bridging hardware and application through algorithmic approaches and achievable performance metrics.

**Table 3:** Summary of the progress of fatigue and drowsiness detection based on dry electrode EEG.

Ref.	Year	Device	Channels	Model	Research Area	Category	Performance
[93]	2019	Muse2016	4	SVM/KNN/DT/ LDA/QDA	Drowsiness	2	subject-independent: 86.50%
[72]	2020	Cognionics	24	Graph theory	Fatigue	2	subject-independent: 96.76%
[73]	2020	Cognionics	24	AMCNN-DGCN	Fatigue	2	subject-independent: 95.65%
[76]	2021	OpenBCI	8	Inception-CNN	Drowsiness	2	subject-independent: 95.59%
[94]	2021	OpenBCI	8	Attention-ResNet	Drowsiness	2	93.35%
[95]	2021	Cognionics	24	CNN-Attention	Fatigue PI	2	subject-dependent: Fatigue: 97.80% PI: 98.5%
[96]	2021	Mindo-4	3	BP-AdaBoost	Fatigue	2	subject-dependent: 92.7 ± 0.92% subject-independent: 77.13 ± 0.85%
[97]	2022	Muse2	3	SVM	Drowsiness	2	subject-independent: 78.3%
[74]	2022	Cognionics	24	GLU-Oneformer	Fatigue	2	86.97%
[98]	2022	G-Tec	32	CS+CNN-LSTM	Fatigue	2	CS ratio: 0 → Accuracy: 99.1% CS ratio: 40% → Accuracy: 95% CS ratio: 90% → Accuracy: 92%
[99]	2023	OpenBCI	16	Bagged decision tree	Drowsiness	2	subject-independent: 85.6%
[100]	2023	Self-made	4	MLP/CNN	Drowsiness	2	On device: 96.24% (subject-independent)
[75]	2023	Cognionics	24	SACC-CapsNet	Fatigue	2	Session 1: 94.17% (Subject-dependent) Session 2: 90.59% (subject-dependent) cross-session: 75.86% subject-independent: 71.37%
[76]	2024	Cognionics	18	MASK-3DCNN	Fatigue	4	subject-independent: 94.90%
[101]	2024	DSI-24	24	CS^2^DA	Fatigue	Regression	SEED-VLA: 0.47 (subject-dependent) SEED-VRW: 0.53 (subject-dependent) cross-scenario: 0.6188
[102]	2025	Self-made	2	SGL-Net	Fatigue	4	subject-dependent: 94.10%
